# The Use of Micro‐CT Analysis of the Second Metacarpal to Assess Cortical Bone Loss in Archeological Human Skeletal Remains

**DOI:** 10.1002/ajpa.70209

**Published:** 2026-02-08

**Authors:** Luisa Leiss, Ian Butler, Sophie L. Newman

**Affiliations:** ^1^ School of History, Classics and Archaeology The University of Edinburgh Edinburgh UK; ^2^ Human Anatomy Unit, Biomedical Sciences, School of Infection, Inflammation and Immunology University of Birmingham Birmingham UK; ^3^ School of Geosciences The University of Edinburgh Edinburgh UK

**Keywords:** μ‐CT, cortical bone loss, cortical porosity, cross‐sectional analysis, second metacarpal index

## Abstract

**Objectives:**

The assessment of cortical bone loss in archeological populations can give insights into past lifeways and bone health. The second metacarpal index (MCI) is assessed via radiogrammetry to quantify cortical thickness. However, as this method is limited to a single‐point measurement, this study explores the use of micro‐computed tomography (μ‐CT) to provide additional cross‐sectional parameters as indicators of cortical bone loss.

**Materials and Methods:**

μ‐CT scans were generated for a sample of 46 second metacarpals from two medieval skeletal assemblages from Scotland, UK. Measurement for MCI, cortical area fraction and intracortical porosity were taken at the mid‐point of the diaphysis. They were then assessed for correlation between each parameter and for patterns in bone loss associated with age and sex.

**Results:**

The MCI revealed a gradual decline in cortical thickness with advancing age for both males and females. There was a significantly lower MCI (associated with bone loss) in older individuals compared to younger adults (*p* < 0.05). No significant differences in MCI were detected between the sexes within any age group. A strong positive correlation was identified between MCI and cortical area fraction (*r* = 0.873), while MCI and intracortical porosity percentage had a weak negative correlation (*r* = −0.401).

**Discussion:**

μ‐CT assessment of the second metacarpal allowed for a more extensive cortical analysis, demonstrating that while the MCI is an effective indicator of cortical thinning, assessment of intracortical porosity can provide further insight into the analysis of cortical bone loss within past populations.

## Highlights

- Introduction of an updated protocol to assess the second metacarpal index (MCI) from μ‐CT scans.
- A strong correlation between MCI and cortical area fraction indicates the efficacy of this parameter to assess cortical bone loss.
- Assessment of intracortical porosity reveals variance in bone quality that cannot be captured via assessment of MCI alone.
- A gradual decline in cortical thickness and overall increasing cortical porosity with advancing age was observed in the medieval Scottish sample, but there were no significant differences in cortical bone loss between age‐matched males and females.

## 1 Introduction

Osteoporosis, resulting from a reduction in overall bone mass, is recognized as a significant health issue in the present day (Sözen et al. 2017). It affects a large portion of the elderly population and can lead to increased fracture risk (Brickley and Mays 2019; Kanis et al. 2021). Factors associated with the progression of bone loss include age, biological sex, various diseases, and lifestyle factors (Sözen et al. 2017). While osteoporosis is commonly associated with the increasing aging population today, patterns in bone loss have been researched within archeological populations in a variety of contexts and with different methodological approaches over the past five decades (see review paper by van Spelde et al. (2021) and references therein).

### 1.1 Second Metacarpal Radiogrammetry

The second metacarpal index (MCI) has been previously used in assessing cortical bone loss in past populations (Ives and Brickley 2005). During bone growth and maturation the formation of bone exceeds resorption and thus bone mass increases until a peak is reached, typically in young adulthood (Weaver et al. 2016; Šromová et al. 2023). With advancing age this reverses and bone resorption exceeds bone formation, resulting in a reduction in bone density, mass and a general degeneration of the bone microstructure and mechanical properties (Eastell et al. 2016; Sözen et al. 2017). This progresses from trabecular to cortical bone and leads to a trabecularisation of the endosteal surface of the cortical bone, causing cortical thinning (Osterhoff et al. 2016). Detecting these changes in cortical bone architecture allows the analysis of bone loss throughout a given population. Radiogrammetry of the second metacarpal enables quantification of cortical bone thickness based on the proportion (%) of cortical bone to the total width of the bone at the diaphyseal midpoint of the second metacarpal (Western and Bekvalac 2020). Various studies have used the MCI to investigate patterns in cortical bone loss in archeological populations aligned to biological sex and age, as well as between populations, and in comparison to modern clinical data (e.g., Mays 1996; Glencross and Agarwal 2011; Beauchesne and Agarwal 2014; Curate et al. 2019; Western and Bekvalac 2020). There is a general consensus between these studies that there is a decrease in cortical thickness with advancing age. Many have further identified that older females (OF) have the lowest MCI values, which might be linked to the effects of menopause (Mays 1996; Curate et al. 2019). However, these patterns have been found to vary over time and space, instilling caution in assumptions regarding biological sex, age, and cortical bone loss (Agarwal 2012, 2021). Therefore, cortical thickness has been interpreted as a general indicator for bone quality and health in association with past living conditions (Beauchesne and Agarwal 2014; Agarwal 2021). However, this approach is limited to relying on a single‐point measurement that cannot detect degenerative changes within the cortical tissue, such as porosity.

### 1.2 μ‐CT Analysis of the Second Metacarpal

In addition to changes at the endosteal surface associated with bone loss, the porosity within the cortical bone itself increases, which is mainly attributed to a widening of Haversian canals and increased presence of resorption cavities (Osterhoff et al. 2016; Ott 2018). These changes of the bone microanatomy can be assessed by obtaining the cortical area fraction (cortical area/total area (Ct.Ar/Tt.Ar)) and the intracortical porosity (cortical pore area/cortical area (Ct.Po)), which are both well‐established histomorphometric parameters, calculated as a proportional percentage similar to the MCI (Bouxsein et al. 2010).

Micro‐computed tomography (μ‐CT) is a non‐destructive imaging technique where a sample is rotated in the path of X‐rays to acquire projection images at regularly spaced angles (Gössl et al. 2006). Individual two‐dimensional slices are then reconstructed from the projection images and can be combined to create a three‐dimensional model (volume) of the scanned sample (Boerckel et al. 2014). Due to its spatial resolution of up to a few microns, μ‐CT can be used to assess changes in trabecular and cortical bone microstructure (Bouxsein et al. 2010). Nishiyama et al. (2010) for example found increased intracortical porosity and cortical thinning in osteoporotic women using this approach, while Chen and Kubo (2014) found a relationship between cortical porosity and fracture risk at the femoral neck as assessed via CT imaging. Regarding the second metacarpal, there have been two major publications using a μ‐CT approach. Lazenby et al. (2008) explored the trabecular structure at the head and base of the second metacarpal, finding evidence for a more robust architecture at the distal epiphysis linked to biomechanical loading (Lazenby et al. 2008). They further reported age‐related changes in female individuals within the sample (Lazenby et al. 2008). Stock et al. (2022) found that trabecular bone volume fraction of second metacarpals was lower in OF, corresponding well to their estimated age (Stock et al. 2022). Further, the qualitative assessment of cortical porosity from mid‐diaphyseal cross‐sections revealed increased intracortical porosity in older individuals (Stock et al. 2022). Despite these advances in the investigation of bone loss in the second metacarpal via μ‐CT analysis, a means to quantify the cortical changes observed has yet to be explored. Although research investigating cortical parameters such as porosity with μ‐CT exists (e.g., Palacio‐Mancheno et al. 2014), the focus seems to be on the trabecular architecture. This could be due to the fact that classical histological approaches are still the norm for detailed cortical morphometry analysis due to clearer tissue contrasts and better resolutions (Chavassieux and Chapurlat 2022). Only very recently García‐Martínez et al. (2023) have shown that μ‐CT can provide results very similar to those achieved by traditional histology which offers the possibility to move away from destructive techniques to analyze archeological bone.

This study aims to further develop the methodological approach for the assessment of bone loss via the MCI using the imaging technique of μ‐CT, and to introduce the additional cross‐sectional parameters of cortical area fraction and intracortical porosity to allow a more detailed cortical analysis. These methods will be applied via a pilot study to a medieval Scottish population to explore how patterns aligned to age and biological sex can potentially be explored within larger population studies to consider intrinsic and extrinsic influences on cortical bone loss.

## 2 Materials and Methods

### 2.1 Skeletal Sample

This study includes a sample of individuals from two Scottish medieval skeletal collections, Ballumbie and St. Andrews, which have been permanently housed at the School of History, Classics and Archeology (HCA), University of Edinburgh for the purposes of teaching and research since their excavation (see below). Ethical approval for this study was obtained from the Research Ethics Committee for the HCA (University of Edinburgh) prior to beginning the research. Excavations at Ballumbie, in the north‐east of the city of Dundee, Scotland, in 2005 resulted in the recovery of the skeletal remains of approximately 200 individuals (Hall and Cachart 2005). Radiocarbon dating has revealed that the site was in use from the 6th to 17th centuries (Canmore 2005; Willows 2016). Following excavation within the former graveyard of the Holy Trinity Church in St Andrews in 1989–1991 and 2003, the remains of about 200 individuals were recovered, and are likely representative of a period of cemetery usage between 1410 and 1620 (Rees et al. 2008). The individuals of both populations are believed to be representative of the lay population of their respective contexts (Rees et al. 2008; Willows 2016). As both populations further represent relatively similar geographic and temporal backgrounds, and as MCI values from both sites did not differ significantly (*p* = 0.661), they were pooled and treated as one sample in this study to achieve a more representable sample size for the purposes of this pilot study (see 4.1 for further discussion).

A sample of individuals from each assemblage was identified based on the presence of a second metacarpal. Second metacarpals with damage resulting in the exposure of the medullary cavity were excluded to minimize taphonomic influences that could result in an alteration of the peri‐ and endosteal surfaces (Ives and Brickley 2004). Metacarpals from the left side were preferentially selected unless they were absent or too fragmented, in which case they were substituted with the right side. This was done to ensure sampling consistency to facilitate reproducibility of the method and comparability with previous studies. Further, the procedural guidelines from Ives and Brickley (2004) acknowledge that side does not affect the MCI analysis and there does not seem to be a link to handedness (Vehmas et al. 2005; Reid et al. 2008). A total of 46 metacarpals were sampled from 37 individuals from the Ballumbie collection and 9 individuals from the St Andrews collection.

Biological sex was estimated for each individual within the study sample by scoring the morphology of the pelvis and cranium using Phenice (1969) and Buikstra and Ubelaker (1994), respectively. Individuals of indeterminate sex or where the most sexually dimorphic elements of the pelvis and skull were absent have not been included. For estimating age, the morphology of the pubic symphysis (Brooks and Suchey 1990) and the auricular surface (Lovejoy et al. 1985) were examined. Dental attrition was scored using Brothwell (1981). Individuals were divided into three age groups: young adults (YA) = 18–29 years, middle adults (MA) = 30–49 years and older adults (OA) = 50+ years (Table 1). This follows an age classification system that is well established in archeological MCI bone loss research (e.g., Mays 1996; Glencross and Agarwal 2011; Beauchesne and Agarwal 2014) and ensures comparability to other studies.

**TABLE 1:** Total sample size split by age (YA = young adults, MA = middle adults and OA = older adults) and sex.

Age category	YA (18–29 years)		MA (30–49 years)		OA (50+ years)		Total	
	*n*	%	*n*	%	*n*	%	*n*	%
Female	8	17.4	14	30.4	3	6.5	25	54.3
Male	7	15.2	8	17.4	6	13.1	21	45.7
Combined	15	32.6	22	47.8	9	19.6	46	100

For a more detailed analysis these categories will be further divided into young females (YF), young males (YM), middle adult females (MF), middle adult males (MM), OF and older males (OM).

### 2.2 Micro‐CT

All second metacarpals were scanned using the X‐ray μ‐CT scanner located at the Experimental Geoscience Facility of the University of Edinburgh. The instrument consists of a Feinfocus 10–160 keV dual transmission and reflection X‐ray source, a Micos ultra high precision air‐bearing rotating sample table and a Perkin Elmer XRD0822 20 × 20 cm 1‐megapixel amorphous silicon flat panel x‐ray camera which has a terbium doped gadolinium oxysulfide scintillator.

**FIGURE 1:**
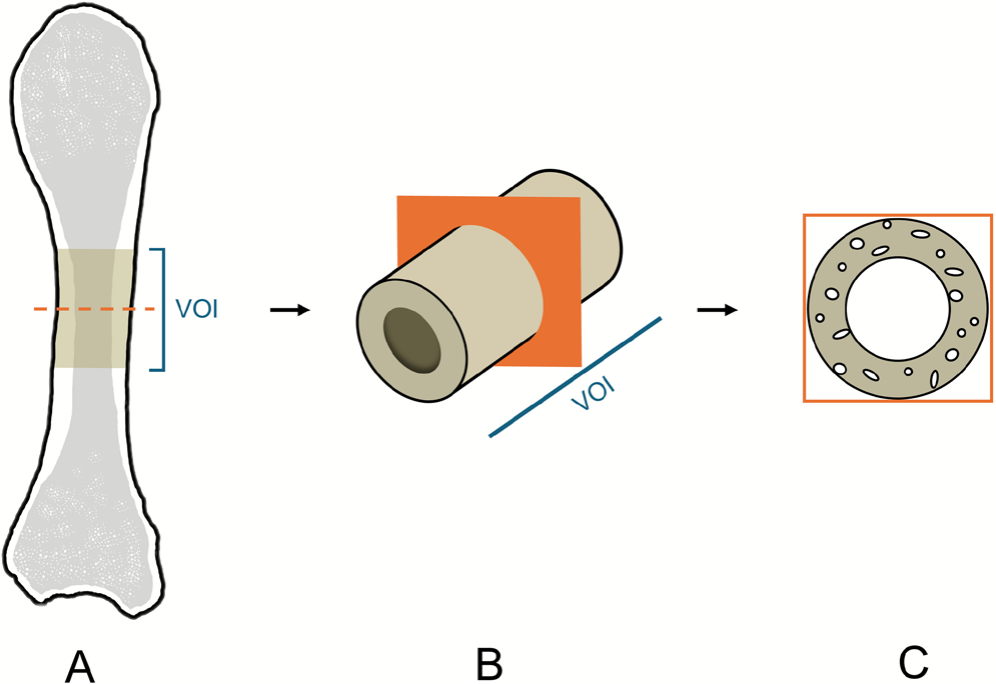
(A) Whole bone scan. (B) Diaphyseal volume of interest (VOI) scan of (C) tomographic slices of higher resolution for cross‐sectional analysis.

Full bone scans of each metacarpal were taken (Figure 1A) using X‐rays with 120 keV peak energy, with 25 W target power loading. During a full 360° rotation, 1200 projections of 1 s exposure were collected, and reconstructed by means of filtered back projection resulting in tomographic slices with a voxel size of 88.9 μm. Additionally, a 1.2 cm long volume of interest (VOI) around the metric midshaft of each metacarpal was scanned using the same settings to achieve high resolution (voxel size of 13.9 μm) cross‐sectional tomographic slices of the midshaft (Figure 1B,C). The midpoint of each bone was aligned with the X‐ray camera's horizontal centre line by means of a laser level.

### 2.3 MCI Protocol

All measurements to calculate the MCI were taken in *3D Slicer (Version 5.6.2)* (Fedorov et al. 2012) using the μ‐CT volumes of each whole bone (see Supplementary File 1 for detailed protocol).

**FIGURE 2:**
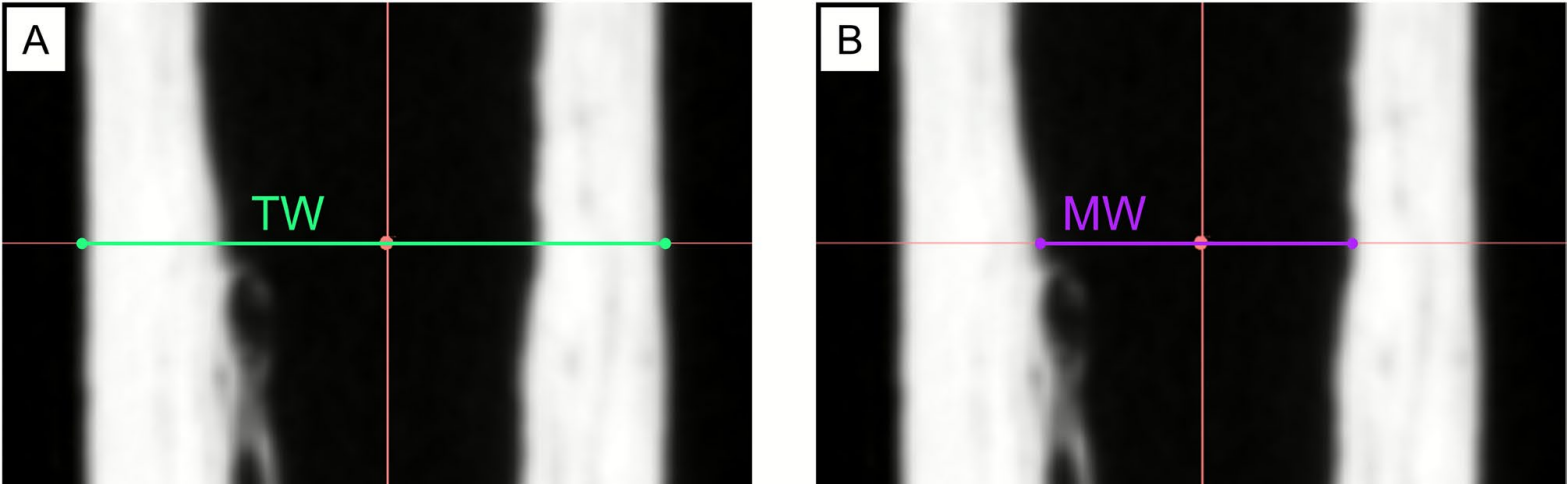
Annotated MCI measurements taken at the coronal midpoint slice. (A) Total width (TW) and (B) Medullary width (MW).

First, a linear transformation was performed to align the palmar surface of the metacarpal 3D render with the anterior side of the region of interest (ROI) box to ensure correct orientation. This view then represents the anteroposterior (AP) position of the metacarpal as used in the traditional radiographic MCI method (Brickley and Mays 2019). Next, the ROI box was adjusted to the maximum length of the bone to identify the metric midpoint. Subsequently, the centre of the ROI box was adjusted to the centre of the medullary cavity of the bone by using the cross‐sectional slice in the axial view panel. Coronal and sagittal planes were then aligned with this point (steps 2–4 in Supplementary File 1). Finally, the MCI measurements of total bone width (TW) and medullary width (MW) were taken for each metacarpal in the coronal section plane along the ROI box midpoint as shown in Figure 2.

All measurements were recorded in mm and the MCI in percentage (%) was calculated to two decimal places for each individual using the following equation:

$$\mathrm{MCI}=\frac{\left(\mathrm{Tw}-\mathrm{Mw}\right)}{\mathrm{Tw}}\times 100$$

Following the procedural guide of Ives and Brickley (2004), bony spurs were in‐ or excluded in the measurement depending on whether they were complete (attached to the cortex at two points) or broken (attached only at one point).

### 2.4 Cross‐Sectional Analysis

**FIGURE 3:**
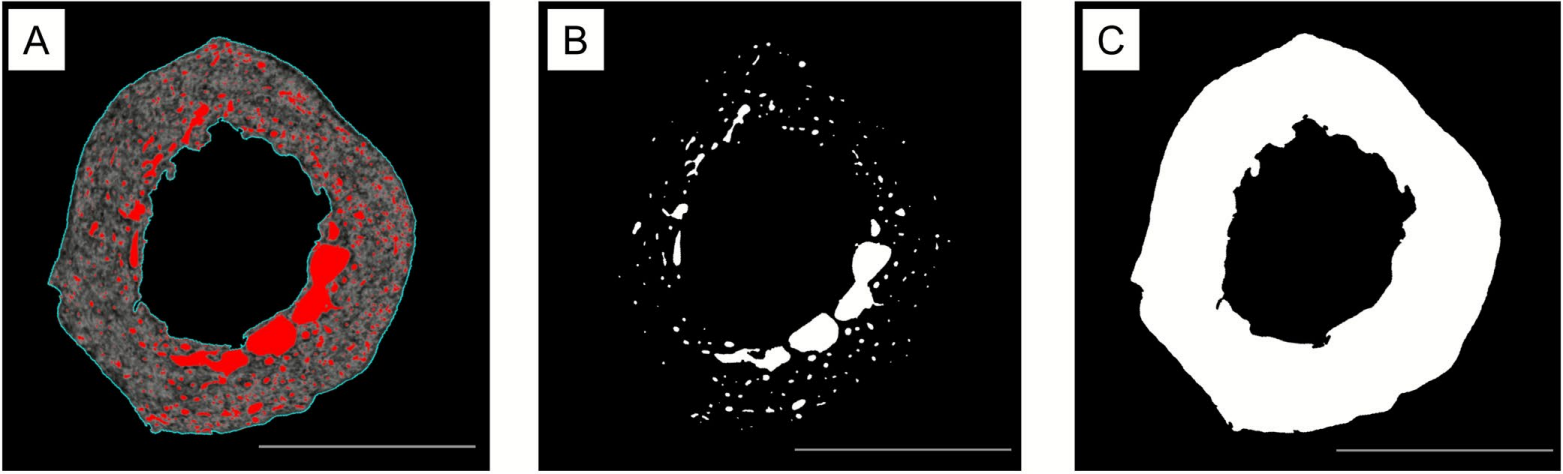
(A) Automatic pore selection using the lumen thresholding (red spaces) and outlining of the cortex (blue lines). (B) Final pore selection after manual modification. (C) Isolated cortical area. Palmar is up. Scale bar = 5 mm.

Cortical area fraction (Ct.Ar/Tt.Ar) and intracortical porosity (Ct.Po) were obtained from the midpoint slice of the high‐resolution diaphysis μ‐CT volumes. The cross‐sectional slices were analyzed using the *Pore Extractor 2D* plugin toolkit in *ImageJ* (Cole et al. 2022). This toolkit was designed for the analysis of histological sections rather than μ‐CT slices, thus some image modifications were necessary. First, the colors of the tomographic slices were inverted to create a white background which is what the toolkit recognizes as pore space. Subsequently, the slice was processed following the recommended steps and settings outlined in Cole et al. (2022). Within the “pore extractor” setup a pore lumen threshold of 161 was selected as this seemed to accurately identify the pore spaces across all cross‐sections (Figure 3A). The pore border threshold was not applied as the pore borders could not be successfully isolated by this tool which, according to the authors, occurs in dark colored images which is the case for the inverted μ‐CT slices (Cole et al. 2022). Bright outliers with a radius of two pixels or less were removed to automatically delete falsely selected areas along the peri‐ and endosteal borders. Further, wrongly selected areas were removed and missing pores added manually to obtain optimum results (Figure 3B,C). Trabecularised pores were included in the later analysis as they are associated with age related changes (Osterhoff et al. 2016). Cracks and/or gaps along the periosteal border that could be associated with taphonomic processes and diagenetic alterations based on the descriptions and examples provided by Booth et al. (2016) and Kendall et al. (2018) were filled in to avoid their selection as additional pore space or exclusion from the cortical area isolation. This affected a total of seven individuals.

The resulting percentage of intracortical porosity generated is an approximation of the true porosity, providing an overall indication of the relative porosity to achieve comparability between individuals.

### 2.5 Statistical Analysis

All statistical analysis was performed in *Past (Version 4.14)* (Hammer et al. 2001) and the significance level for all test outputs was set to *α* = 0.95. Normality of all datasets was assessed using a Shapiro–Wilk test (see Supplementary File 2). A two‐way ANOVA considering age and sex was performed to test for statistically significant differences between group means, as well as for a possible interaction of these variables with the MCI and Ct.Ar/Tt.Ar (Hammer 2024). Depending on the output of the normality tests, a one‐way ANOVA or Kruskal‐Wallis test was performed to assess differences in each parameter between the age groups of each sex. A Tukey's or Dunn's post hoc test was applied when significant differences were identified. Correlation analysis was performed to assess the presence and strength of a potential relationship between Ct.Ar/Tt.Ar and MCI (Pearson's *R* test), as well as between Ct.Po and the MCI (Spearman's Rank test).

Intra‐ and inter‐observer reliability for the MCI recording method was assessed by the primary author and a participant with no prior experience in the use of the software by measuring a sub‐sample of the second metacarpals (*N* = 15 for intra‐ and *N* = 10 for inter‐observer testing) and calculating the coefficient of reliability (*R*) for the MCI where *R* > 0.95 indicates a very good reliability (Goto and Mascie‐Taylor 2007). The intra‐observer results indicate only minimal measurement errors and thus a very good reliability (*R* = 0.98). The inter‐observer results show greater deviations with a coefficient of reliability of *R* = 0.85.

## 3 Results

All data supporting the following results can be found in Supplementary File 3.

### 3.1 MCI Assessment by Age and Sex

**FIGURE 4:**
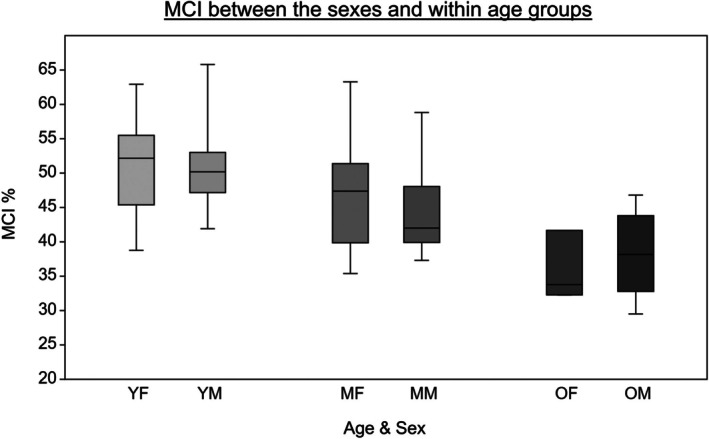
MCI distribution across the sexes per age group. MF, middle adult females; MM, middle adult males; OF, older females; OM, older males; YF, young females; YM, young males.

Overall, there is a steady decrease in MCI values between the age groups, with the YA having the greatest average cortical thickness and the OA the lowest (Table 2 and Figure 4). Male and female MCIs are very similar, with the female MCI mean (*x�* = 46.85%) only slightly higher than the male mean (*x�* = 44.88%), while the range of the male MCI values (36.30%) is slightly wider than the female range (31.00%). The highest mean MCI values were seen in young adult males (*x�* = 51.30%; albeit very similar to those of young adult females at *x�* =50.93%), and the lowest values were seen in older adult females (*x�* = 35.91%: Table 2 and Figure 4).

**TABLE 2:** Summary statistics of the MCI analysis for each group in % (except *n*).

Group		Sample size (*n*)	Mean (*x�*)	Standard deviation (SD)	Range	
					Minimum	Maximum
YA	Female	8	50.93	7.37	38.76	62.93
	Male	7	51.30	7.39	41.91	65.81
	Total	15	51.10	7.11	38.76	65.81
MA	Female	14	46.86	7.93	35.39	63.28
	Male	8	44.27	6.87	37.30	58.82
	Total	22	45.92	7.50	35.39	63.28
OA	Female	3	35.91	5.04	32.28	41.66
	Male	6	38.22	6.19	29.51	46.80
	Total	9	37.45	5.62	29.51	46.80
All	Female	25	46.85	8.51	32.28	63.28
	Male	21	44.88	8.39	29.51	65.81
	Total	46	45.95	8.42	29.51	65.81

A two‐way ANOVA test revealed statistically significant differences in MCI between the age groups (*F*(2,40) = 9.629, *p* ≤ 0.001) but not between the sexes (*F*(2,40) = 0.09159, *p* = 0.764). No statistically significant interaction between age and sex was found (*F*(2,40) = 0.3885, *p* = 0.681). Following a Tukey's post hoc test, the OA (*x�* = 37.45%, SD = 5.62) were found to have significantly lower MCIs when compared to the YA (*x�* = 51.10%, SD = 7.11, *p* ≤ 0.001) and MA (*x�* = 45.20%, SD = 7.50, *p* = 0.01). No statistically significant difference was found between the MCIs of the YA and the MA (*p* = 0.091). Both sexes were explored individually with regards to the detected age‐related differences.

Within the female sample, there was a continuous decline of mean cortical thickness with advancing age. The ranges in MCI for the YF and MF are very similar while the OF present a smaller range and a considerably lower mean MCI (Table 2 and Figure 4). There were significant differences in MCIs between the three age groups (*F*(2,22) = 4.345, *p* = 0.026). The MCIs of the OF (*x�* = 35.91%, SD = 5.04) were found to be significantly lower compared to those of the YF (*x�* = 50.93%, SD = 7.37, *p* = 0.02). The MCIs did not differ significantly between the YF and MF (*p* = 0.455) and between the MF and OF (*p* = 0.079).

There was a steady decline in MCIs with advancing age among the male sample. This decline seemed more gradual as the difference in mean MCI between the YM and MM was similar to that between the MM and OM (Table 2 and Figure 4). There were significant differences in the MCIs between the three age groups (*F*(2,18) = 5.907, *p* = 0.011). The MCIs of the OM (*x�* = 38.22%, SD = 6.19) were found to be significantly lower than those of the YM (*x�* = 51.30%, SD = 7.39, *p* = 0.008). The MCIs did not differ significantly between the young‐ and MM (*p* = 0.146) and between the MM and OM (*p* = 0.259).

### 3.2 Cross‐Sectional Area

There is a steady decrease in cortical area fraction at the second metacarpal midshaft from the young adult to the older adult age groups, similar to the overall MCI distribution, as seen in Table 3. This pattern can be observed within both female and male samples (Table 3). The young and older adult males have a greater average cortical area (YM *x�* = 73.12% and OM *x�* = 62.62%) compared to the females in these age groups (YF *x�* = 71.99% and OF *x�* = 58.10%) while the MF present a similar mean cortical area to the MM (MF *x�* = 67.72% and MM *x�* = 67.33%). While the mean cortical area for both sexes in the middle adult age group is very similar, females in this age group have a considerably wider range compared to the males (MF = 30.37% and MM = 15.96%). Within the younger and older age groups the males tend to have greater ranges than the females.

**TABLE 3:** Summary statistics for Ct.Ar/Tt.Ar in each group in % (except *n*).

Group		Sample size (*n*)	Mean (*x�*)	Standard deviation (SD)	Range	
					Minimum	Maximum
YA	Female	8	71.99	4.46	65.18	77.73
	Male	7	73.12	7.37	63.02	86.06
	Total	15	72.51	5.79	63.02	86.06
MA	Female	14	67.72	8.62	50.36	80.73
	Male	8	67.33	3.68	60.05	76.01
	Total	22	67.57	7.63	50.36	80.73
OA	Female	3	58.10	3.68	54.89	62.12
	Male	6	62.62	5.95	57.62	71.56
	Total	9	61.11	5.54	54.89	71.56
All	Female	25	67.93	8.05	50.36	80.73
	Male	21	67.91	7.48	57.62	86.06
	Total	46	67.92	7.71	50.36	86.06

Similar to the MCI analysis, a two‐way ANOVA revealed statistically significant differences only between the age groups (*F*(2,40) = 7.905, *p* = 0.001). Within the sex groups, significant differences were found between young and older female (*p* = 0.024) and male individuals (*p* = 0.024) (see Supplementary File 4 for detailed outputs).

**FIGURE 5:**
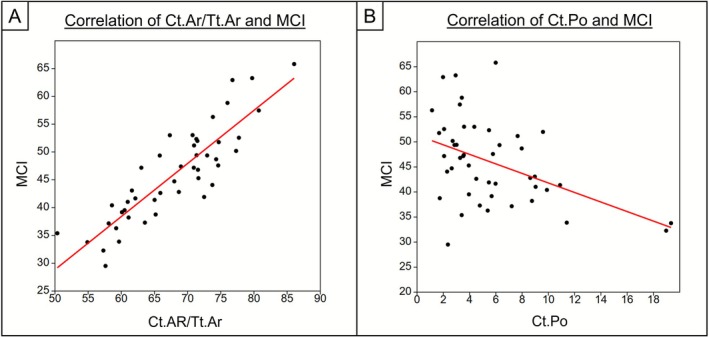
Relationship between (A) Ct.Ar/Tt.Ar and MCI and (B) Ct.Po and MCI. Red line shows the line of best fit.

There was a very strong positive correlation between Ct.Ar/Tt.Ar and the MCI (Pearson's R: *r*(44) = 0.873, *p* < 0.001) (Figure 5A). About 76% of variation is explained by the model (*r* ^*2*^ = 0.762, *F =* 140.87, *p* < 0.001), indicating that the MCI increases with increasing cortical area.

### 3.3 Intracortical Porosity

The average percentage of intracortical porosity increases with advancing age, with the lowest mean porosity seen in YA (*x�* = 3.49%) and the highest seen in OA (*x�* = 9.20%: Table 4). Young adult females have a lower mean porosity (*x�* = 2.30%) compared to young adult males (*x�* = 4.86%), while middle and older adult females have higher mean porosities (MF *x�* = 5.88% and OF *x�* = 14.76%) compared to the males of these age groups (MM *x�* = 5.21% and OM *x�* = 5.44%). The range in intracortical porosity values is similar for the young and middle adult age groups, but considerably greater for the OA.

**TABLE 4:** Summary statistics for Ct.Po in each group in % (except *n*).

Group		Sample size (*n*)	Mean (*x�*)	Standard deviation (SD)	Range	
					Minimum	Maximum
YA	Female	8	2.30	0.89	1.14	3.58
	Male	7	4.86	2.02	2.06	7.97
	Total	15	3.49	1.97	1.14	7.97
MA	Female	14	5.88	2.64	2.94	9.89
	Male	8	5.21	3.08	2.29	10.89
	Total	22	5.64	2.75	2.29	10.89
OA	Female	3	14.76	7.61	5.98	19.34
	Male	6	6.42	3.38	2.35	11.40
	Total	9	9.20	6.25	2.35	19.34
All	Female	25	5.80	4.79	1.14	19.34
	Male	21	5.44	2.80	2.06	11.4
	Total	46	5.64	3.97	1.14	19.34

Following a Kruskal‐Wallis test for group comparison, no significant differences in porosity were identified between the male age groups (*p* > 0.05). However, for females the porosity of the young individuals was significantly lower compared to that of the middle (*p* = 0.004) and older (*p* = 0.001) age groups (see Supplementary File 4 for detailed outputs).

There was a weak to moderate negative correlation between intracortical porosity and the MCI (Spearman's Rank: *r*_*s*_(44) = −0.401, *p* = 0.006) (Figure 5B). This indicates that as porosity increases, the MCI tends to decrease.

## 4 Discussion

### 4.1 Age and Sex Related Bone Loss in MCI

Similar average MCI value ranges were observed by Beauchesne and Agarwal (2014) in an Italian Imperial Roman population, by Mays (2006) in Romano‐British women, as well as by Mays (1996) in the English medieval Wharram Percy population. Thus, the MCI ranges obtained from the μ‐CT scans are consistent with those obtained by the traditional radiographic assessment and can be compared to previous studies. The similarity of the MCI range to other archeological populations also indicates that the peak bone mass of the medieval Scottish individuals lies clearly below the averages of more modern samples such as Virtama and Helelä (1969) or Haara et al. (2006) which have been frequently used for comparison (e.g., Mays 1996; Western and Bekvalac 2020).

The results relating to age‐related bone loss follow an expected trend of a steady decline in cortical thickness from the younger to older adult age groups. This was also observed within the male and female samples. This is a pattern that has been observed in other archeological populations (e.g., Mays 2006; Beauchesne and Agarwal 2014). However, while there appeared to be significant differences in cortical bone loss parameters associated with age, there were no significant differences between males and females, and sex and age were not found to have a significant interaction. This is similar to the results reported by Beauchesne and Agarwal (2014) who found no significant interaction between sex and MCI, as well as for age‐sex interaction in their sample of Imperial Roman individuals.

**FIGURE 6:**
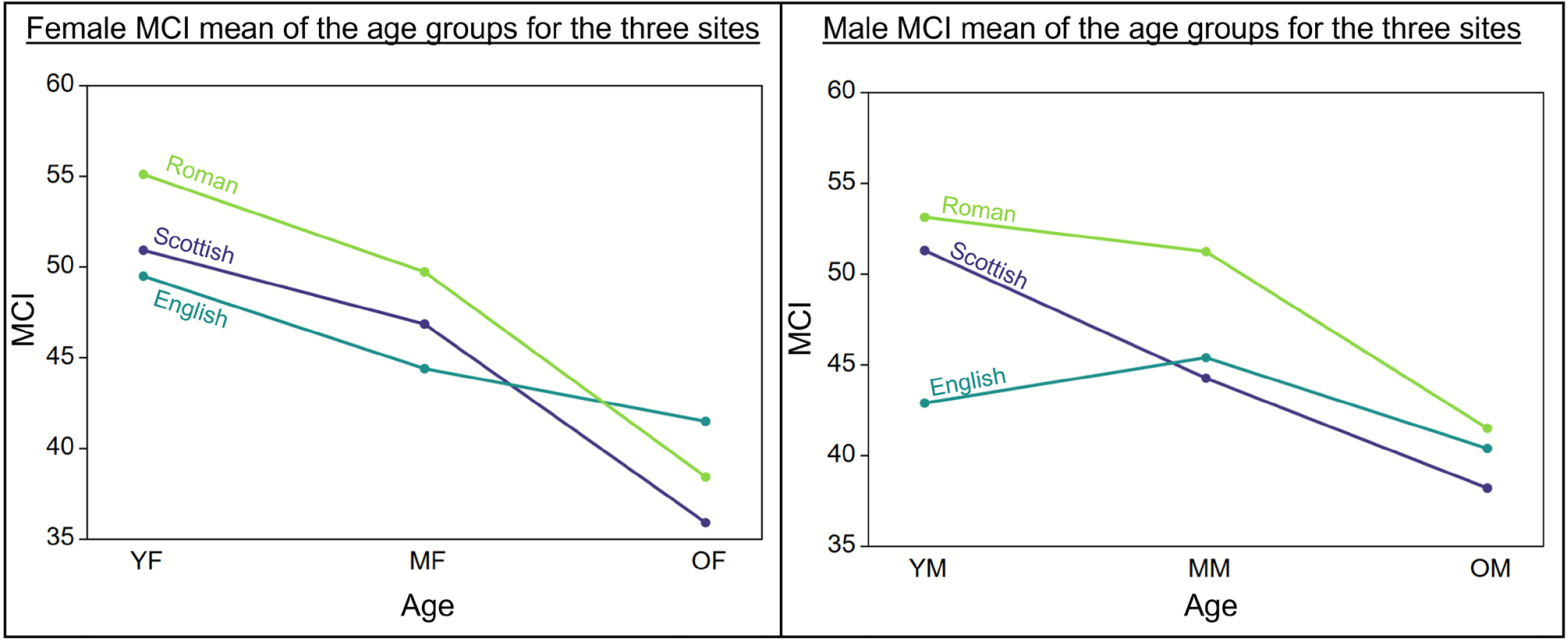
Average MCI comparison for the age groups of females and males of the present study (Scottish), Wharram Percy (English) (Mays 1996) and Velia (Roman) (Beauchesne and Agarwal 2014).

Ekenman et al. (1995) also reported similar average MCI for medieval Swedish males and females. Mays (1996) reported a steady decline in MCI with age for the females of Wharram Percy, though the current study presents an even greater decline from the MF to the OF (Figure 6). However, the male sample of the current study shows a different pattern, presenting a steady decline in MCI where Mays (1996) showed only slight variation between the male age groups with an average MCI peak in the middle old age group (Figure 6). Although the urban Imperial Roman population from Velia studied by Beauchesne and Agarwal (2014) represents a different environment and time, their observed bone loss pattern is very similar to that of the current study sample as both males and females show a steady decline in cortical thickness with increasing age (Figure 6). However, their average MCI values for each age and sex group were higher than those of the Wharram Percy sample and the investigated Scottish sample (Beauchesne and Agarwal 2014).

Beauchesne and Agarwal (2014) suggest biocultural differences including diet and activity as possible causes for the differences seen between their Roman sample and Wharram Percy. Mays (1996) explained that both men and women had a physically demanding agricultural‐focused lifestyle in rural medieval England with little task‐based gendered labor division. Based on this, and the absence of obvious risk factors such as severe malnutrition or other metabolic diseases, Mays (1996) concluded that the observed sex differences are primarily due to postmenopausal bone loss rather than lifestyle. Though less documentary evidence describing the medieval Scottish lifestyle exists, rural life was also centred around agricultural work while in urban settlements the trade industry was dominant (Ewan 2012; Whyte 2012; Ide 2023). Following the analysis of activity patterns of medieval Scottish individuals, Ide (2023) found that while a task‐based gendered labor division was present, medieval Scottish men as well as women carried out strenuous physical work. Thus, it can be assumed that the males and females within this sample had a similar physically demanding lifestyle that might be reflected in the comparable bone loss patterns.

Ballumbie and St Andrews have been previously described as populations of lower socioeconomic status based on the prevalence of diseases and dietary analysis (Ide 2023; Willows 2016). Stable isotope analysis by Willows (2016) found that the diets of individuals from Ballumbie and St Andrews were similar and represent a typical medieval diet mainly consisting of plant‐based terrestrial resources. This diet was further influenced by the seasonality of agriculture, potentially even causing chronic undernutrition which would have had an adverse effect on bone modeling during childhood growth (Beauchesne and Agarwal 2014; Willows 2016). This might explain the lower average cortical thickness that is commonly observed in archeological populations when compared to modern clinical data. Interestingly, Mays (1996) did not report any deficiencies in the Wharram Percy individuals while their average cortical thicknesses are similar, and for some groups even lower than those of the present Scottish sample.

Contrary to several other MCI studies (e.g., Mays 1996; Glencross and Agarwal 2011; Beauchesne and Agarwal 2014), the young adult female sample within the current study did not present a higher average MCI than the males, which is often associated with acquiring earlier peak bone mass, possibly due to the metabolic demands for pregnancy (Mays 1996; Glencross and Agarwal 2011; Beauchesne and Agarwal 2014). This could indicate that males and females both acquired their peak bone mass before the age of 30 years in this sample. One rather unusual pattern observed in the present analysis was that the MF had a slightly higher mean MCI than the MM. This pattern has not been described by other MCI studies, and it is unknown what this could reflect. One possible explanation might simply be the unequal sample size distribution within that age group where the female group contained almost twice as many individuals as the male group. It should also be noted that the Ballumbie individuals comprise about 80% of the studied sample and express a relatively low degree of sexual dimorphism with almost 50% of the individuals being placed within “probable” categories for sex estimation. To what extent this might be reflected in the similarities in bone loss patterns should be further investigated. Beauchesne and Agarwal (2014) explain that strong physical activity might help to maintain bone mass throughout age, causing differences between the age and sex groups to be less distinct than it might be seen in a modern sample.

While the older adult female MCIs were lower than those of the older adult males, no significant differences were found in the mean MCI between these groups. Considering modern clinical osteoporosis studies, a difference between the sexes would be expected between the oldest age groups associated with postmenopausal osteoporosis (Kanis et al. 2021). Large scale MCI data comparing the sexes of modern populations is limited as bone mineral density (BMD) analysis is the standard mean of assessment in the clinical context today (LeBoff et al. 2022). However, Curate et al. (2019) reported significantly higher averages in the male MCIs and a faster decline in the female MCIs of their more modern 19th–20th century population. A stronger decline in the female MCI was also present in the 20th century sample of Virtama and Helelä (1969). Again, the observed pattern might be connected to lifestyle factors but could also be related to the smaller sample size of the OA and the fact that the exact age of death of these individuals remains unknown, making it unclear if postmenopausal osteoporosis had manifested in the form of cortical thinning (Mays 1996). Focus of this study was the introduction of the additional cross‐sectional parameters and the results are supportive of the need to further explore these potential patterns within a larger population study to permit more robust interpretations regarding lifestyle and bone loss within Scottish medieval populations.

### 4.2 Cross‐Sectional Analysis

Cortical area fraction and intracortical porosity were investigated as new parameters as they are common variables assessed within the cortical bone microstructure and are linked to age‐related changes (Bouxsein et al. 2010; Agnew and Stout 2012). As with the MCI, they are both relative measures reported in %, allowing for a more size‐independent analysis.

**FIGURE 7:**
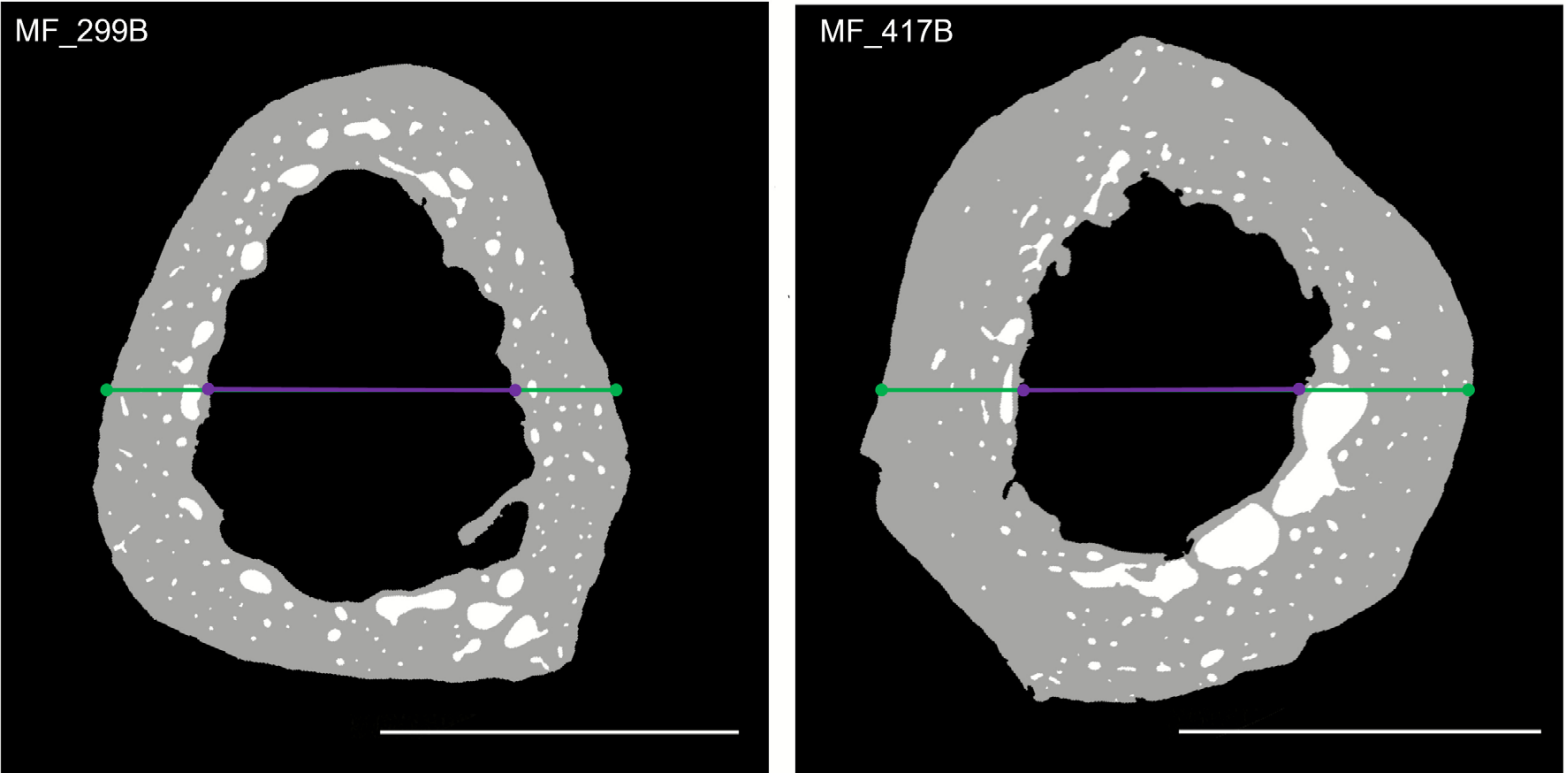
Two middle adult females with similar Ct.Po but different MCI and Ct.Ar/Tt.Ar values. Lines show MCI measurement points. This includes a complete spur due to a trabecularised pore in the second tomographic slice. Palmar is up. Scale bar = 5 mm.

The results of the cortical area analysis presented the expected pattern of a gradual thinning of the cortical bone with progressing age, showing almost the exact same pattern overall and within the age and sex groups as the MCI results. This was further confirmed by the strong positive correlation that showed that as the cortical area increases, the MCI also increases. This observation indicates that the single point‐based MCI alone is a good indicator in detecting these patterns at the metacarpal diaphysis. However, neither the MCI nor the cortical area assessment considers intracortical pore spaces (Zebaze et al. 2009; Agnew and Stout 2012). The study by Agnew and Stout (2012) investigating intracortical porosity and its contribution to bone loss in human ribs found that there was an average of 4% difference in cortical area fraction when excluding the porosity in their sample. Thus, individuals with a similar cortical area might present different degrees of intracortical porosity and therefore different bone strengths, which cannot be detected by only investigating either cortical thickness or area fraction (see Figure 7). This shows that if only one of these parameters is considered, the resulting patterns might vary considerably from those detected by the other variables. The example in Figure 7 shows two MF with similar intracortical porosity percentages but very different MCI and cortical area values. The second tomographic slice in Figure 7 further shows the impact the inclusion of a bony spur can have, as this notably decreases the medullary width and therefore increases MCI.

**FIGURE 8:**
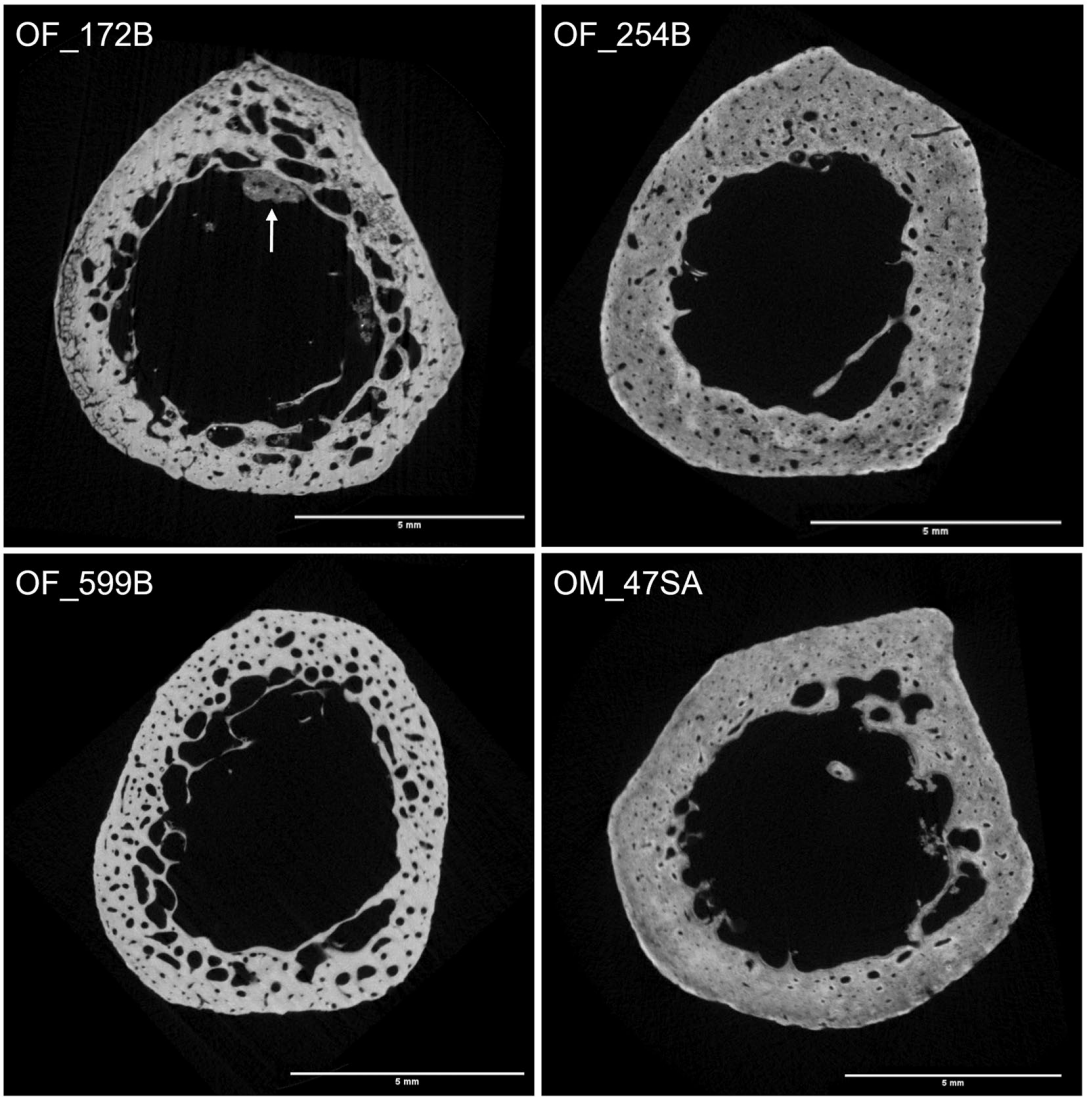
Left top and bottom: Older females with highest intracortical porosity (Soil ingress visible at OF_172B as indicated by white arrow. This has been removed prior to analysis as explained by protocol in Section 2.4). Right top: Older female with porosity more similar to middle old group average. Right bottom: Average older male porosity example. Palmar is up. Scale bar = 5 mm.

The results of the porosity percentage analysis generally show the expected trend of an increase in intracortical porosity while the cortical thickness (as assessed by MCI) decreases. This increase in porosity can be observed across the age groups overall and within both sexes, but the correlation analysis has shown that this relationship is rather weak. There is considerably more variation in porosity within the individual sex groups and the age groups therein. Studies investigating the femur and tibia have shown that the intracortical porosity increases more steadily in males while females show a more rapid increase from middle to older age groups (Thomas et al. 2005; Nirody et al. 2015). A similar pattern was observed within the metacarpals of this study, as significant differences in porosity were identified only in the female sample cohort. Most notable were two individuals who presented a considerably higher intracortical porosity than the rest of the sample, both individuals being older adult females. As seen in Figure 8, these extremes are unlikely due to measurement error as the high porosity can be observed from the midpoint slice. Considering the small sample size of only three OF, these two individuals make up two‐thirds of this group, which might explain the great difference seen in average intracortical porosity between the OF and OM. The third older adult female individual had a porosity percentage more comparable to the middle adult group or the older adult male average as shown in Figure 8.

Due to the loose definition of the older age group as 50+ years it is not known whether the two females were considerably older than the remaining older adult individuals. A bigger sample size would be needed to explore this further, but the clear difference in average cortical porosity between older males and females could be an indicator for the effect of postmenopausal bone loss, as these two individuals were also at the lower end of the MCI and cortical area values, indicating overall low bone mass.

Considering all three assessed parameters (MCI, Ct.Ar/Tt.Ar and Ct.Po) together, a young male stood out by presenting the overall highest MCI and largest cortical area, but also one of the greatest intracortical porosities among the YM. This individual had a thick metacarpal cortex, but the analysis also showed numerous trabecularised pores of considerable size that were included in the MCI and area fraction analysis, increasing those values. Average porosity in the YM was about twice as high as that of the YF, mainly due to the YM having greater proportions of trabecularised pores. Nirody et al. (2015) found that the YF in their study had lower total pore area and lower pore numbers throughout the tibial cortex than their YM, but this was not discussed further. Further, Thomas et al. (2005) reported higher porosities for their YM in the femur diaphysis when compared to females. Whether this could be linked to different growth and development timings, including the achievement of peak bone mass, between the sexes in the second metacarpal should be further investigated.

Methodologically it was possible to quantify cortical area fraction and intracortical porosity from the μ‐CT scans using the *ImageJ* plugin; however, small pores such as Haversian canals were difficult to detect. Chavassieux and Chapurlat (2022) mention that for μ‐CT scans this is due to resolution differences when compared to traditional histology. Haversian canals make up a considerable amount of pore space within the cortex, and Wang and Ni (2003) state that their widening is responsible for the majority of age‐related intracortical porosity changes. Thus, it would be ideal to confidently incorporate smaller pores for future analyses; however, as expected trends (i.e., general increase in porosity with age) could be detected across the individual groups, the present analysis is sufficient for comparing the intracortical porosity to the MCI as well as to cortical area fraction. Cortical area fraction is a commonly used direct measurement of bone mass (Peck and Stout 2007), and thus the strong positive correlation with the MCI supports the argument that the MCI is a reliable method to quantify bone loss. Zebaze et al. (2009) and Agnew and Stout (2012), however, both suggest that intracortical porosity should be included in the bone loss investigation, especially when considering bone fragility, as not only cortical thinning but also increased intracortical porosity enhances fracture risk (Bjørnerem 2016).

### 4.3 Applicability of μ‐CT Scans for Assessment of Cortical Bone Loss

Part of this study was to establish a protocol to obtain MCI from μ‐CT scans. This was achieved by using freely available open‐source software that is frequently used to explore μ‐CT data and bone microarchitecture (e.g., Agnew and Stout 2012; Çakmak et al. 2019; Cole et al. 2022). This ensures easy accessibility and reproducibility of the protocol. MCI results of the current project were not compared to cut‐off points and/or used to diagnose osteoporosis. For assessing bone loss patterns at the population level, the overall pattern is of greater interest than individual values.

Only minimal intra‐observer error was detected, indicating reliable repeatability. The inter‐observer error was slightly higher, indicative of the influence of user experience in the use of the software and application of the recording protocol in the repeatability of the method. Ives and Brickley (2004) also reported significant differences between observers in the recording of MCI and recommended defining standardized measurement positions, such as the differentiation between complete and incomplete endosteal bony spurs, which were adopted into the present protocol. As the present study is a first attempt to obtain the measurements from μ‐CT scans, the protocol seems to achieve similar results as the traditional method, proving to be useful in detecting cortical bone loss at the second metacarpal.

Regarding the μ‐CT approach, future research should focus on moving from the 2D to a 3D assessment. While Peck and Stout (2007) described the cross‐sectional area measurements of cortical bone as a reliable and accurate indicator of bone mass, the analysis of a 3D volume might reveal more detail. The approach of Gilmour et al. (2021) to quantify cortical area along the standardized DXR ROI at the metacarpal diaphysis could be expanded to obtain the cortical volume using high resolution μ‐CT scans. This would allow for a comparison of the MCI to area and to a volumetric measurement.

### 4.4 Study Limitations

As mentioned throughout the discussion, the biggest limitation for interpreting the observed bone loss patterns in the current project was that of the sample size and the distribution within the estimated age and sex groups.

With regards to sample sizes in bioarcheology it should be considered that skeletal assemblages represent only a small subset of the people who lived and subsequently died at the site at a given time (Waldron 2007). This subset can further be reduced due to poor preservation of the remains, especially with regards to older individuals (Waldron 2007; Newman et al. 2023). This may affect a higher proportion of OF, where bone loss due to postmenopausal osteoporosis and lifestyle factors generates a greater susceptibility to taphonomic processes, thus creating a preservation bias (Walker 1995; Gowland 2007; Newman et al. 2023). However, assumptions should not be made regarding norms for patterns in cortical bone loss aligned to sex and age for all temporal and geographic contexts (Agarwal 2012, 2021).

In this pilot study sample almost 50% of the individuals were categorized as MA, while OA constituted only 20% of the sample, despite being the age group of greatest interest in association with investigating bone loss. This is a known limitation within bioarcheology and Martrille et al. (2007) stated that there is a tendency to overestimate the age‐at‐death of young individuals while underestimating the age‐at‐death of older individuals with common aging methods, resulting in a larger middle adult age group. In the middle adult age group, there were almost twice as many females as males (14F and 8M), whereas in the older adult group there were more males than females (3F and 6M). These unequal numbers might not be representative of the true population and might distort the results, causing them to be less powerful and conclusive in the wider context (Waldron 2007). It must also be noted that in this study, as well as in many of the cited MCI studies, biological age is estimated and might not reflect the actual chronological age of the individuals (see Couoh 2017). Reliance on age and sex estimations limits the interpretative power of the patterns observed by the MCI and suggested additional parameters. Thus, future research should include application of this protocol to a larger sample of individuals of known age and sex, ideally encompassing greater numbers of older individuals, to validate the correlations noted between the three parameters. Despite this current limitation, it must be acknowledged that the observed MCI patterns generally follow the expected trends associated with age‐related bone loss, and as this updated method is likely to be implemented more broadly in the analysis of assemblages where age and sex is by necessity estimated, the continuation of the use of broad age groupings as in existing MCI studies is recommended to allow comparability within biological age‐matched samples.

Considering additional further research potential of this updated protocol and its application within bioarcheological research, fragility fractures have been frequently assessed in conjunction with the MCI, often in an attempt to directly identify or diagnose osteoporosis (e.g., Mays 1996, 2000, 2006; Glencross and Agarwal 2011; Beauchesne and Agarwal 2014; Curate et al. 2019). Additionally, other pathological changes that might affect bone remodeling rates and therefore the cortical thickness and/or porosity, such as vitamin D deficiency, should be considered. This might give further insights into the overall skeletal health and consequences of bone loss. Indeed, Beauchesne and Agarwal (2017) recommend using a multi‐method approach of assessing multiple skeletal sites with different methodologies to achieve a more complete overview of bone loss and health over the course of life. Therefore, a wider analysis encompassing μ‐CT analysis and macroscopic indicators of skeletal pathology would be recommended to better understand patterns associated with cortical bone loss in Scottish Medieval assemblages and archeological populations more broadly.

## 5 Conclusion

A preliminary study of the combination of parameters for MCI, cortical bone area and porosity as measured via μ‐CT revealed potential patterns in age and sex related bone loss in the pooled Scottish medieval sample. A comparison to the existing literature of similar geographic location and time revealed some similarities but also differences connected to various factors, possibly including lifestyle. The argument that medieval cortical bone loss resembles the modern trend with postmenopausal osteoporosis as the driving factor as proposed by previous research (e.g., Mays 1996) cannot be confirmed for this study sample as the males also showed significant bone loss with advancing age. However, a larger scale study is required before these patterns can be validated, with this study serving to demonstrate the potential of the updated methodological approach in exploring bone loss in archeological contexts.

This project demonstrates that μ‐CT derived cross‐sections of cortical bone have the potential to be analyzed using established histomorphometric parameters. Future research should attempt to acquire even higher resolution scans to enable a detailed two‐ and three‐dimensional analysis, and also seek application to a documented skeletal collection where age and sex are known. The importance of intracortical porosity in bone loss analysis and the usefulness of imaging modalities such as μ‐CT have gained more recognition in clinical research over the years, but less within osteoarcheology (e.g., Chen and Kubo 2014; Lerebours et al. 2015; Molino et al. 2020). Considering the importance of incorporating intracortical porosity as evaluated in the present study, and as cortical bone is less prone to diagenetic changes, the analysis of intracortical porosity together with the measures of cortical thickness and/or area might provide an alternative to assessing BMD at sites of fragility fracture risk in archeological bone (Nishiyama et al. 2010; Chen and Kubo 2014; Tripp et al. 2018; Rasmussen et al. 2019).

## Author Contributions

**Luisa Leiss:** writing – original draft, methodology, conceptualization, visualization, funding acquisition, formal analysis, writing – review and editing. **Ian Butler:** writing – review and editing, methodology, software, resources. **Sophie L. Newman:** conceptualization, funding acquisition, writing – review and editing, supervision, resources.

## Funding

This research was funded by the Archeology Research Support Fund, School of History, Classics and Archeology (University of Edinburgh).

## Ethics Statement

Ethical approval for this research was obtained from the Research Ethics Committee of the School of History, Classics and Archeology (University of Edinburgh). The authors adhered to the guidelines set out by the British Association of Biological Anthropology and Osteoarcheology (BABAO) on the handling and 2D/3D digital imaging of archeological human remains.

## Conflicts of Interest

The authors declare no conflicts of interest.

## Supporting information


**Data S1:** Supporting Information.

## Data Availability

The data that supports the findings of this study are available in the Supporting Information of this article.
