# Supplementary material for: The Use of Micro‐CT Analysis of the Second Metacarpal to Assess Cortical Bone Loss in Archeological Human Skeletal Remains
Source: Am J Biol Anthropol. 2026 Feb 8;189(2):e70209. doi: 10.1002/ajpa.70209 (PMC12884138; doi:10.1002/ajpa.70209)
Supplement: Supplementary file 1 — Data S1: Supporting Information. [file AJPA-189-e70209-s001.docx]

SUPPLEMENTARY FILE 1:

Protocol for measuring the second metacarpal index (MCI) from µ-CT scans using *3D Slicer* (Version 5.6.2)

1.
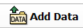
Importing µ-CT data:
   - Go to “**Add Data**” ( ) and select the file
   - Tick the “**Show Options**” box in the dialogue field, then untick the “**Single File**” box and click **OK**
   -
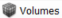
Go to the “**Volumes**” module ( ), click on “**Volume Information**” and copy the first/second “**Image Spacing**” value into the third box
     *(in this project this value was 0.08893mm)*
   -
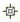
Click on “**Center view**” ( ) in all planes to bring scan back into focus
2. Bringing bone into correct plane *(not necessary if bone already is in AP/PA view)*
   - Go to the “**Data**” module (
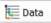
 ) and to the tab “**Transform hierarchy**”
   - Right click on the file name and select “**Insert transformation**”
   - Go back to the “**Subject hierarchy**” tab and right click on the cube next to the file name (
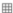
). The linear transformation should be highlighted in yellow.
   -
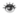
Go to the “**Volume Rendering**” module (
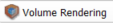
 ) and click on the eye symbol ( ) next to “Volume” and “Display ROI”
   -
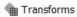
Go to the “**Transforms**” module ( ) and use the “**Rotation**” controls to bring the bone in the correct plane (*here= palmar view*)
     - Bring ROI box into correct orientation by clicking on the “A” for anterior in the blue 3D render box controls
     -
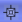

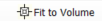
When the bone moves out of the field of view, go back to “**Volume Rendering**” and click on “**Fit to Volume**” ( ), as well as on
        in the blue 3D render box

-
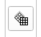
When bone is oriented correctly, go to “**Apply transform**” within the “Transforms” module, select the file name and “**Harden transform**” ( )
- Result of re-orientation:


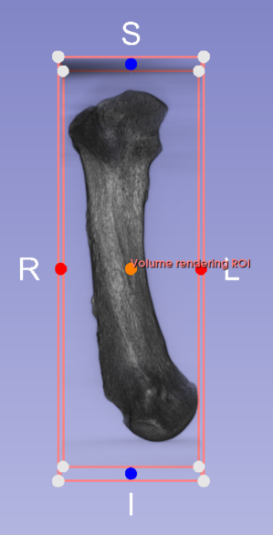

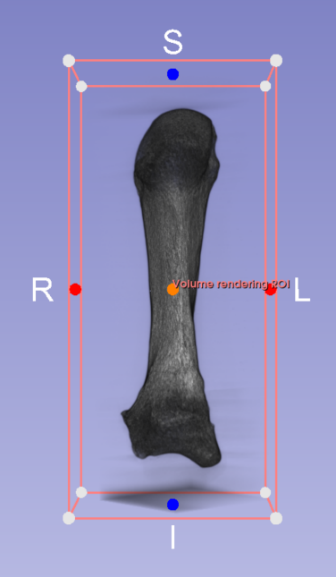
Before: After:

1. Adjusting ROI box to maximum length

- Go to the **coronal view** (green box) and adjust ROI box to the maximum bone length by scrolling through the slices:


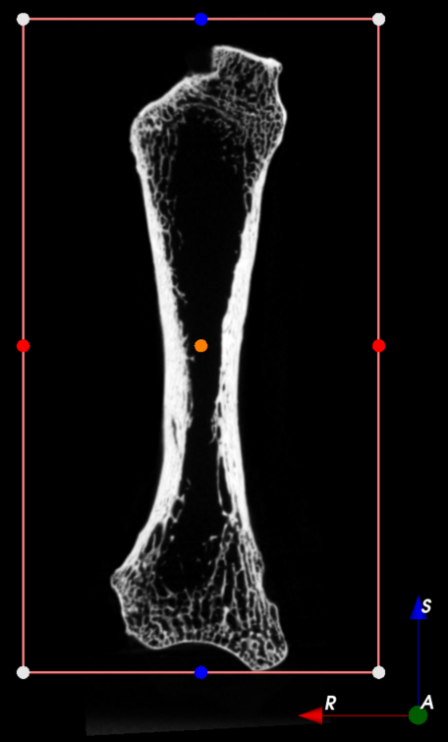

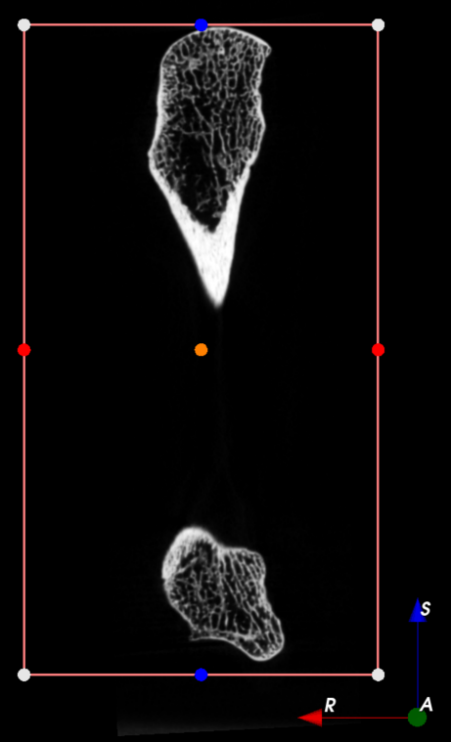
 most superior point: most inferior point:

1.
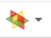
Finding the midpoint

- Click on to show how the planes intersect
-
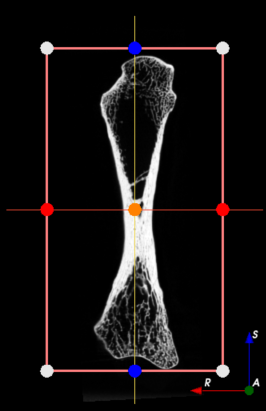

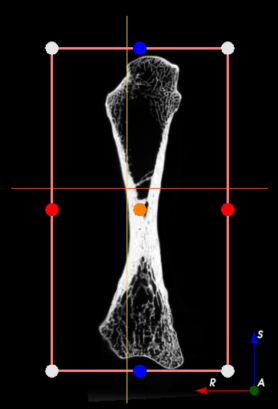
Bring axial plane (red line) to the midpoint by clicking on the orange point at the centre of the ROI box in the coronal plane view:
- Centre “Volume rendering ROI” point into the middle of the cross-section:


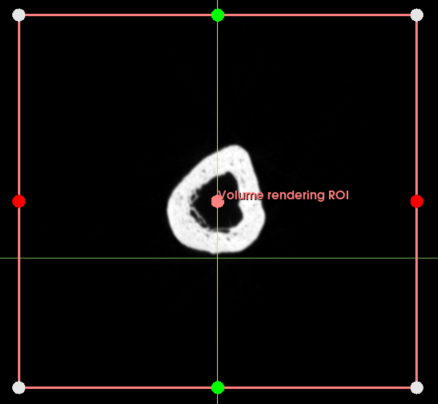

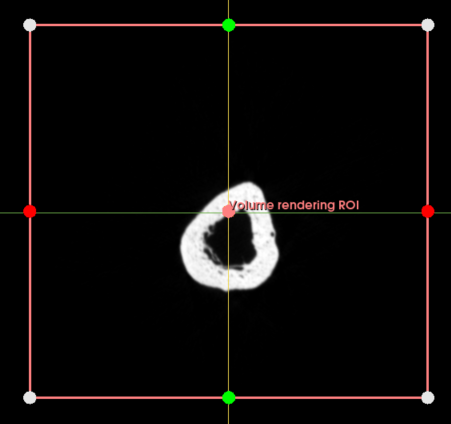
ROI point off-centre on cortex: ROI point centred:


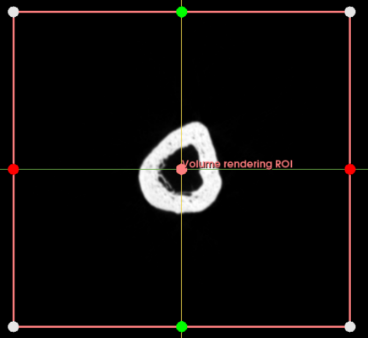


- Adjust other planes by clicking on the “**Volume rendering ROI**” point in the axial plane *(The text “Volume rendering ROI” should now appear in each view)*

1. Taking measurements

- Go to the “**Markups**” module (
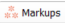
 ) and select “**Line**” (
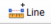
 )
- For better orientation: draw a horizontal and a vertical line through the ROI box (*length of vertical line equals maximum bone length*)


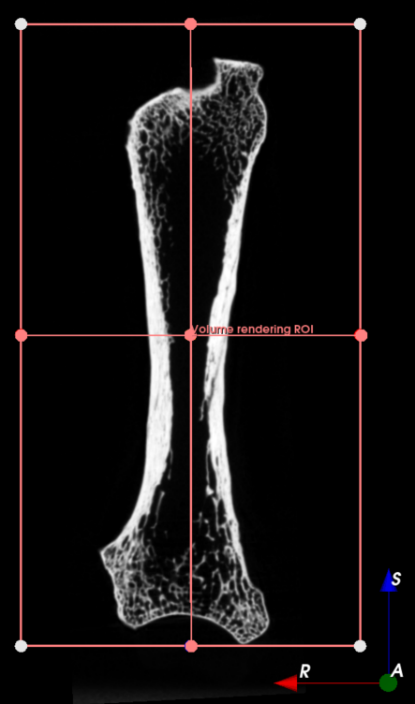

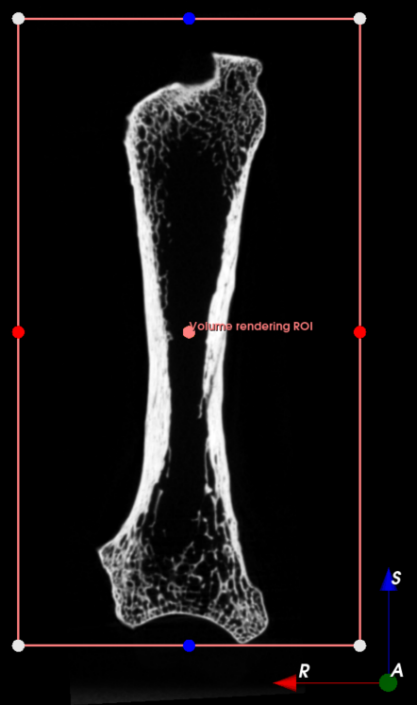
 without guides: with guiding lines:

- Measure total width (TW) and medullary width (MW) along horizontal guide


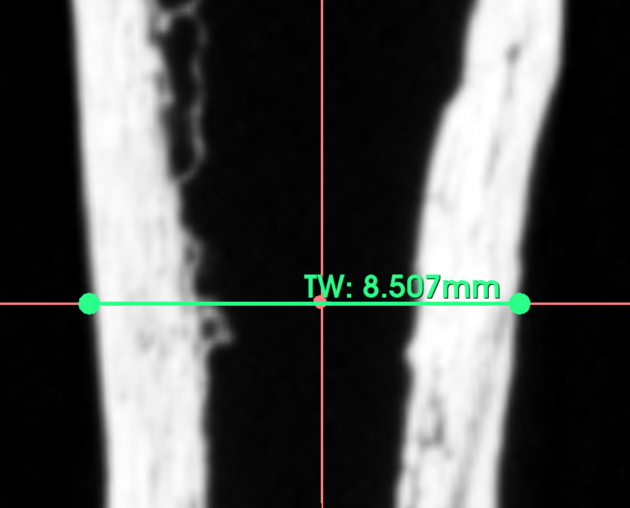


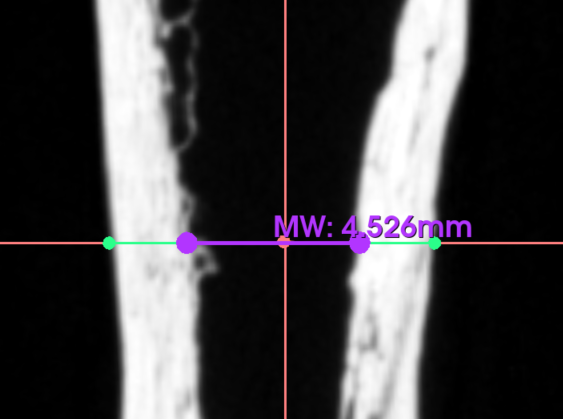


*(Glyph and text size can be reduced for accurate measuring. Enlarged here for illustration purposes only)*

SUPPLEMENTARY FILE 2:

Normality test (Shapiro-Wilk) outputs for the individual subsamples of the MCI analysis:

| *Sub- sample* | ***Sample size (n)*** | ***Shapiro-Wilk w*** | ***p-value*** |
| --- | --- | --- | --- |
| ***Ballumbie*** | 37 | .978 | .666 |
| ***St Andrews*** | 9 | .909 | .307 |
| ***Females total*** | 25 | .975 | .769 |
| ***Males total*** | 21 | .972 | .765 |
| ***YA*** | 15 | .960 | .683 |
| ***MA*** | 22 | .951 | .324 |
| ***OA*** | 9 | .970 | .889 |
| ***YF*** | 8 | .983 | .975 |
| ***MF*** | 14 | .966 | .820 |
| ***OF*** | 3 | .897 | .286 |
| ***YM*** | 7 | .900 | .331 |
| ***MM*** | 8 | .848 | .091 |
| ***OM*** | 6 | .995 | .998 |

Normality test (Shapiro-Wilk) outputs for the whole sample of the cortical area fraction (Ct.Ar/Tt.Ar) and intracortical porosity analysis (Ct.Po):

| *Analysis* | ***Sample size (n)*** | ***Shapiro-Wilk w*** | ***p-value*** |
| --- | --- | --- | --- |
| ***Ct.Ar/Tt.Ar*** | 46 | .984 | .749 |
| ***Ct.Po*** | 46 | .821 | <.001 |

SUPPLEMENTARY FILE 3:

| ***Sample Information*** | | | | |  | ***MCI measurements*** | | |  | ***Cross-sectional analysis*** | |
| --- | --- | --- | --- | --- | --- | --- | --- | --- | --- | --- | --- |
| ***Collection*** | ***SK no.*** | ***Sex*** | ***Age*** | ***Side*** |  | ***TW*** | ***MW*** | ***MCI*** |  | ***Ct.Ar/Tt.Ar*** | ***Ct.Po*** |
| Ballumbie | 42 | F | MA | L |  | 7.51 | 4.64 | 38.22 |  | 61.10 | 8.75 |
| Ballumbie | 64 | F? | MA | L |  | 6.32 | 4.08 | 35.39 |  | 50.36 | 3.4 |
| Ballumbie | 67 | M? | MA | R |  | 9.02 | 5.29 | 41.38 |  | 65.02 | 10.89 |
| Ballumbie | 72 | M? | MA | R |  | 8.89 | 5.10 | 42.63 |  | 65.89 | 4.51 |
| Ballumbie | 135 | F? | YA | L |  | 7.06 | 3.32 | 53.03 |  | 70.75 | 3.58 |
| Ballumbie | 153 | F | YA | L |  | 7.99 | 4.42 | 44.72 |  | 67.99 | 2.64 |
| Ballumbie | 172 | F? | OA | L |  | 8.11 | 5.50 | 32.28 |  | 57.30 | 18.97 |
| Ballumbie | 225 | M | YA | L |  | 8.29 | 2.84 | 65.81 |  | 86.06 | 5.99 |
| Ballumbie | 234 | M? | YA | L |  | 7.58 | 3.78 | 50.19 |  | 77.31 | 2.71 |
| Ballumbie | 254 | F? | OA | L |  | 7.47 | 4.36 | 41.66 |  | 62.12 | 5.98 |
| Ballumbie | 299 | F | MA | L |  | 6.80 | 4.05 | 40.41 |  | 58.59 | 9.89 |
| Ballumbie | 417 | F | MA | R |  | 8.20 | 3.94 | 51.99 |  | 71.47 | 9.59 |

Data supporting the results: Demographic information of the studied sample (Key: F= female, F?= probably female, M?= probably male, M= male; YA= young adult of 18-29 years, MA= middle adult of 30-49 years, OA= old adult of 50+ years; L= left, R= right), MCI measurements (TW= total width, MW= medullary width) and cross-sectional analysis of cortical area fraction (Ct.Ar/Tt.Ar) and intracortical porosity (Ct.Po). *All raw measurements are rounded and reported to two decimal places here.*

| Ballumbie | 419 | M? | OA | L |  | 8.85 | 5.06 | 42.81 |  | 68.68 | 8.62 |
| --- | --- | --- | --- | --- | --- | --- | --- | --- | --- | --- | --- |
| Ballumbie | 432 | F | YA | L |  | 7.73 | 4.74 | 38.76 |  | 65.18 | 1.73 |
| Ballumbie | 435 | F? | MA | R |  | 8.18 | 3.00 | 63.28 |  | 79.73 | 2.94 |
| Ballumbie | 501 | F | MA | L |  | 7.43 | 4.23 | 43.07 |  | 61.60 | 8.97 |
| Ballumbie | 512 | F | YA | R |  | 7.24 | 3.75 | 47.39 |  | 68.99 | 3.56 |
| Ballumbie | 521 | F? | YA | L |  | 7.08 | 2.62 | 62.93 |  | 76.76 | 1.98 |
| Ballumbie | 522 | M? | MA | L |  | 10.10 | 4.15 | 58.82 |  | 76.01 | 3.41 |
| Ballumbie | 525 | F | MA | L |  | 7.77 | 4.25 | 45.29 |  | 71.63 | 3.94 |
| Ballumbie | 567 | M | MA | R |  | 8.80 | 5.19 | 41.03 |  | 60.97 | 9.03 |
| Ballumbie | 599 | F | OA | L |  | 7.97 | 5.28 | 33.78 |  | 54.89 | 19.34 |
| Ballumbie | 606 | M? | MA | L |  | 8.13 | 4.92 | 39.53 |  | 60.50 | 3.95 |
| Ballumbie | 613 | M | YA | L |  | 9.31 | 5.41 | 41.91 |  | 72.49 | 5.47 |
| Ballumbie | 623 | M | YA | L |  | 7.72 | 3.68 | 52.33 |  | 71.32 | 5.48 |
| Ballumbie | 628 | M | YA | L |  | 9.26 | 4.89 | 47.17 |  | 63.02 | 2.06 |
| Ballumbie | 657 | M? | YA | L |  | 8.94 | 4.59 | 48.69 |  | 74.30 | 7.97 |
| Ballumbie | 682 | M | MA | L |  | 7.85 | 4.39 | 44.07 |  | 73.73 | 2.29 |
| Ballumbie | 706 | F | MA | L |  | 6.97 | 3.68 | 47.17 |  | 70.93 | 3.52 |
| Ballumbie | 721 | F? | YA | L |  | 7.23 | 3.49 | 51.77 |  | 74.69 | 1.68 |
| Ballumbie | 725 | M | OA | L |  | 8.51 | 4.53 | 46.80 |  | 71.56 | 3.28 |

| Ballumbie | 761 | M? | OA | L |  | 8.65 | 6.10 | 29.51 |  | 57.62 | 2.35 |
| --- | --- | --- | --- | --- | --- | --- | --- | --- | --- | --- | --- |
| Ballumbie | 784 | M? | MA | L |  | 10.01 | 6.28 | 37.30 |  | 63.56 | 4.78 |
| Ballumbie | 790 | M | MA | L |  | 9.19 | 4.65 | 49.38 |  | 72.95 | 2.85 |
| Ballumbie | 810 | F | MA | L |  | 7.05 | 3.00 | 57.46 |  | 80.73 | 3.26 |
| Ballumbie | 834 | M? | YA | L |  | 7.90 | 3.71 | 53.01 |  | 67.32 | 4.36 |
| Ballumbie | 848 | F | MA | L |  | 6.89 | 3.49 | 49.41 |  | 71.35 | 3.01 |
| St Andrews | 26 | F? | YA | L |  | 7.99 | 3.49 | 56.30 |  | 73.81 | 1.14 |
| St Andrews | 27 | F? | MA | R |  | 7.11 | 4.53 | 36.29 |  | 59.23 | 5.38 |
| St Andrews | 42 | F? | MA | L |  | 8.14 | 3.97 | 51.17 |  | 70.97 | 7.66 |
| St Andrews | 46 | M? | OA | L |  | 7.80 | 5.16 | 33.88 |  | 59.66 | 11.4 |
| St Andrews | 47 | M? | OA | L |  | 8.92 | 5.61 | 37.16 |  | 58.10 | 7.21 |
| St Andrews | 57 | F? | YA | L |  | 7.75 | 3.68 | 52.56 |  | 77.73 | 2.06 |
| St Andrews | 60 | F? | MA | L |  | 8.91 | 4.67 | 47.58 |  | 74.61 | 5.78 |
| St Andrews | 61 | F? | MA | L |  | 6.78 | 3.43 | 49.36 |  | 65.80 | 6.28 |
| St Andrews | 69 | M? | OA | R |  | 8.00 | 4.86 | 39.17 |  | 60.10 | 5.68 |

| *Factor* | ***F-value*** | ***p-value*** |
| --- | --- | --- |
| ***Sex*** | 0.247 | 0.621 |
| ***Age*** | 7.905 | 0.001* |
| ***Interaction*** | 0.366 | 0.700 |

SUPPLEMENTARY FILE 4:

Cortical area fraction (Ct.Ar/Tt.Ar):

Two-way ANOVA output for age and sex groups of Ct.Ar/Tt.Ar.
Significant p-values are indicated with *.

*<0.05

| *Group comparison* | ***p-value*** |
| --- | --- |
| ***YA – MA*** | 0.090 |
| ***YA – OA*** | <0.001* |
| ***MA - OA*** | 0.044* |

Tukey’s post-hoc test output of two-way ANOVA for age group comparisons of Ct.Ar/Tt.Ar. Significant p-values are indicated with *.

*<0.05

ANOVA and Tukey’s post-hoc output for Ct.Ar/Tt.Ar of female age groups. Significant p-values indicated with *:

ANOVA and Tukey’s post-hoc output for Ct.Ar/Tt.Ar of male age groups.
Significant p-values indicated with *:

| ***ANOVA:*** *F*=4.099; *p*=0.031* | |
| --- | --- |
| *Tukey’s post-hoc* | ***p-value*** |
| ***YF – MF*** | 0.388 |
| ***YF – OF*** | 0.024* |
| ***MF – OF*** | 0.111 |

*<0.05

| ***ANOVA:*** *F*=4.271; *p*=0.030* | |
| --- | --- |
| *Tukey’s post-hoc* | ***p-value*** |
| ***YM – MM*** | 0.225 |
| ***YM – OM*** | 0.024* |
| ***MM – OM*** | 0.391 |

*<0.05

Intracortical porosity (Ct.Po):

Kruskal-Wallis and Dunn’s post-hoc output for Ct.Po of male age groups.
Significant p-values indicated with *:

Kruskal-Wallis and Dunn’s post-hoc output for Ct.Po of female age groups.
Significant p-values indicated with *:

| ***Kruskal-Wallis:*** *H*=0.874; *p*=0.646 | |
| --- | --- |
| *Dunn’s post-hoc* | ***p-value*** |
| ***YM – MM*** | 0.973 |
| ***YM – OM*** | 0.408 |
| ***MM – OM*** | 0.412 |

| ***Kruskal-Wallis:*** *H*=13.520; *p*=0.001* | |
| --- | --- |
| *Dunn’s post-hoc* | ***p-value*** |
| ***YF – MF*** | 0.004* |
| ***YF – OF*** | 0.001* |
| ***MF – OF*** | 0.147 |

*<0.05

*<0.05
